# Choking and laryngospasm: Exploring commonalities and treatment strategies

**DOI:** 10.2478/jccm-2025-0010

**Published:** 2025-01-31

**Authors:** Gad Estis, Asia Estis-Deaton, Tiberiu Ezri

**Affiliations:** Herzliya Medical Center, Herzliya, Israel; Assuta Ashdod Hospital, Ashdod, Israel; Kaplan Medical Center, Rehovot, Israel

## To the Editor

Choking is a significant health concern associated with substantial morbidity and mortality. Despite ongoing efforts, an optimal solution remains elusive. We propose that this may stem from an overemphasis on mechanical obstruction as the primary cause. Notably, a foreign body does not always completely occlude the airway. For instance, Saccomanno et al. reported that fish bones were implicated in 67% of choking incidents [1], suggesting that defensive airway reflexes may play a critical role beyond mere mechanical obstruction.

The Expiratory Reflex (ExpR) is a protective mechanism designed to prevent airway obstruction. When a foreign body reaches the larynx and threatens to block the upper airway, an immediate life-saving response is triggered. This response includes breath-holding at any phase of the respiratory cycle, transient laryngeal closure, forceful expiration, and subsequent swallowing or coughing [2].

However, the ExpR can sometimes become improperly activated or “stuck.” Severe choking occurs when the ExpR remains “stuck” in its apnea and forceful expiration phases—a condition resembling a vigorous Valsalva maneuver. For individuals with limited physiological reserves, such as infants, the elderly, or obese patients, this can lead to rapid and severe consequences. This Valsalva-like phenomenon, in our opinion, is a critical factor in choking, explaining why it can be fatal even in the absence of complete airway obstruction. When a foreign body does cause complete obstruction, the situation becomes even more critical.

It is reasonable to assume that physiological mechanisms remain consistent across contexts. However, intriguingly, the phenomenon of the “stuck” ExpR, when observed in the operating theater, is often labeled as laryngospasm, even when triggered by vomiting with solid particles.

Bernard Raymond Fink's pioneering work established the link between laryngospasm and Valsalva-like breath-holding [3]. Subsequent research has primarily focused on laryngeal closure, often overlooking the role of forceful expiration. As a result, the inability to ventilate an intubated patient during laryngospasm is frequently misdiagnosed as bronchospasm, leading to inappropriate treatment.

Given the similarities between choking and laryngo-spasm, it is worth exploring whether treatment strategies effective for one condition could be applied to the other. For example, back blows, the current first-line intervention for choking, aim to dislodge foreign bodies from the larynx. However, could their efficacy extend beyond simply dislodging obstructions?

Research by Al-Metwalli et al. suggests that gentle chest compressions can alleviate extubation laryngo-spasm in children [4]. This raises the possibility that back blows might not only dislodge foreign bodies but also “reset” a stuck ExpR.

**Fig. 1.**
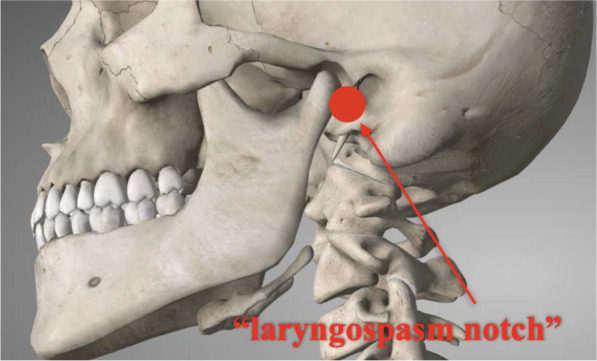
Larson maneuver

Another intervention, the “Larson maneuver,” is a well-established technique for managing laryngo-spasm. It involves applying firm bilateral pressure on the “laryngospasm notch” (Figure 1) while performing a jaw thrust [5]. Considering the parallels between laryngospasm and choking, we propose that this maneuver, proven effective in practice, could also be beneficial for managing choking. Notably, it is simple, accessible, and unique in that it can even be performed by the patient on themselves when choking occurs.

Future research should aim to further elucidate the shared pathophysiology of choking and laryngospasm, as well as refine treatment strategies to improve patient outcomes
